# Autism Spectrum Disorder and Perivascular Spaces: An Integrative Perspective Across the Lifespan

**DOI:** 10.3390/jcm14248886

**Published:** 2025-12-16

**Authors:** Maria Alessandra Sotgiu, Alessandra Carta, Vanna Cavassa, Andrea Montella, Salvatore Masala, Giuseppe Barisano, Stefano Sotgiu

**Affiliations:** 1 Department of Biomedical Sciences, University of Sassari, 07100 Sassari, Italy; montella@uniss.it; 2 Division of Child Neuropsychiatry, Department of Medicine, Surgery and Pharmacy, University of Sassari, 07100 Sassari, Italy; alessandra.carta@aouss.it (A.C.); vanna.cavassa@aouss.it (V.C.); 3 Radiology Unit, Department of Medicine, Surgery and Pharmacy, University of Sassari, 07100 Sassari, Italy; samasala@uniss.it; 4 Department of Neurosurgery, Stanford University, Stanford, CA 94305, USA; barisano@stanford.edu

**Keywords:** autism, perivascular spaces, glymphatic system, aging

## Abstract

Autism Spectrum Disorder (ASD) is a heterogeneous neurodevelopmental condition characterized by persistent social communication difficulties, restricted interests, repetitive behaviors, and frequent medical comorbidities. Although early brain development in ASD has been extensively investigated, its biological progression across adulthood and aging remains largely unexplored. Growing evidence suggests that perivascular space (PVS) abnormalities may indicate impaired neurovascular integrity and reduced glymphatic clearance in ASD. Enlarged perivascular spaces (ePVS) in children commonly present alongside increased extra-axial CSF accumulation and more severe clinical manifestations, consistent with early alterations in CSF homeostasis and neuroimmune signaling. However, whether these abnormalities persist or evolve with aging remains unknown. Given that glymphatic and vascular integrity decline with age, and adults with ASD show elevated rates of sleep, metabolic, and cardiovascular disorders, PVS alterations may represent a unifying mechanism linking early neurodevelopmental divergence with later neurovascular vulnerability and cognitive aging. Advances in ultra-high-field MRI and automated segmentation now enable precise in vivo quantification of PVS burden, offering new opportunities for lifespan studies. By combining structural and functional methodologies, researchers may determine whether PVS constitute enduring traits, dynamic indicators of disease, or actionable therapeutic targets. Understanding their trajectories could provide critical insights into the continuum between neurodevelopmental and neurodegenerative phenomena in autism.

## 1. Introduction

Autism Spectrum Disorder (ASD) is a lifelong neurodevelopmental condition, defined by the Diagnostic and Statistical Manual of Mental Disorders (DSM-5) and the International Classification of Diseases (ICD-11). It is characterized by difficulties in social communication, restricted interests, repetitive behaviours, sensory anomalies, and variable degrees of intellectual disability [1,2].

ASD is highly heterogeneous both between individuals and across development, with features that change over time [3].

Its clinical phenotype is further complicated by multiple comorbidities, spanning neurological and psychiatric conditions, systemic disorders, and sleep disturbances [4,5,6].

Epidemiological studies report a male-to-female prevalence of clinical ASD ratio of 2:1 to 5:1, although diagnostic disparities suggest a narrower actual distribution, particularly among individuals with subtler phenotypes [6].

Etiologically, ASD results from interactions among genetic, epigenetic, and environmental influences, including factors that affect early brain development [7].

Neuroimaging advances over the past two decades have highlighted early alterations in brain structure and function, including increased total brain volume, reduced white matter integrity, abnormal functional and network connectivity, and perfusion abnormalities [8].

These differences evolve across the lifespan: early childhood is often characterized by brain overgrowth, particularly in frontal and temporal regions, while adolescence and adulthood show plateauing or accelerated cortical decline [9].

Although global prevalence is estimated at 1–2% [10,11], population-based rates appear to decrease with age [12]. This decline may reflect reduced life expectancy, driven by comorbidities, accidental injury, and suicide [13], as well as underdiagnosis in older adults due to limited age-appropriate tools, reliance on retrospective development history, and masking of autistic traits through compensatory strategies [14]. As a result, the neurobiological trajectory of ASD beyond childhood remains poorly defined. Key unresolved questions include whether early neural alterations persist, normalize with maturation, or evolve into atypical or premature aging.

Symptom trajectories in adulthood and aging remain poorly characterized. While some studies describe worsening cognitive and behavioural difficulties, others report stability or even improvement [15,16,17].

Neurocognitive findings point to vulnerability in frontal and medial temporal regions–areas critically involved in executive function and memory-whereas evidence of atypical functional connectivity throughout adulthood raises the possibility of premature cognitive aging [18]. Despite these insights, the field still lacks robust and reliable biomarkers capable of tracking neurobiological changes across the autistic lifespan, from early development into older age [19].

Among emerging candidates, perivascular spaces (PVS), also known as Virchow-Robin spaces and first described by Durand-Fardel and later by Virchow and Robin in the mid-19th century [20], have recently attracted increasing interest as potential indicators of neurovascular integrity and glymphatic function [21].

The present review offers an integrative perspective on the role of PVS in ASD. By synthesizing relevant evidence from neuroimaging, neurodevelopmental, and aging research, it outlines possible trajectories of PVS alterations across the lifespan and discusses their implications for clinical practice and future research.

To pursue this aim, we focus on current pediatric evidence, propose a rationale for investigating PVS in later life, and outline future neuroimaging strategies to bridge structural, functional, and clinical domains to advance the understanding of the autistic lifespan.

With this context, the following section introduces PVS.

## 2. Perivascular Spaces: Structural Feature and Functional Role

According to the STRIVE guidelines, PVS are interstitial fluid–filled compartments that accompany penetrating arterioles and venules, but not capillaries, as they traverse the brain parenchyma [22].

Anatomically, they represent expansions of the subarachnoid space, bordered by two basement membranes: the vascular endothelial membrane and the glia limitans formed by astrocytic endfeet [23,24]. A recently described fourth meningeal layer, the subarachnoid lymphatic-like membrane (SLYM), further delineates PVS from the subarachnoid space [25]. This thin barrier plays an important role in facilitating the periarterial influx of newly produced CSF, regulating solute exchange between CSF and the meningeal venous sinus, and supporting meningeal immune activity [26]. Understanding SLYM-related CSF dynamic may be relevant for interpreting PVS alterations in the ASD population. PVS morphology varies by location. In the basal ganglia, arteries are encased by two meningeal layers, whereas cortical regions typically present with a single layer [24].

Physiological forces, including arterial pulsation, vasomotion, respiration, and neural activity, drive convective cerebrospinal fluid (CSF) flow through PVS. CSF enters into the brain parenchyma, mixes with interstitial fluid, exits via perivenous spaces, and drains into meningeal lymphatic vessels [27,28,29].

This bidirectional exchange is facilitated by polarized aquaporin-4 (AQP4) channels on astrocytic endfeet, forming the core of the glymphatic system responsible for interstitial solute clearance and brain homeostasis [30,31]; AQP4 deletion markedly impairs this clearance [27]. Previously identified only through histological examination, PVS can now be visualized in vivo using MRI [31,32]. On scans, they appear linear when visualized along the course of the vessels and parallel to the imaging plane, whereas they appear round or ovoid when the vessel is oriented perpendicular to the imaging plane and seen in cross-sectional view [24]. High-field MRI (≥3T) has enhanced detection even in healthy young individuals, demonstrating that PVS are not inherently pathological [33]. Enlarged (ePVS), defined as >2 mm in diameter, are most commonly observed in the centrum semiovale, subinsular region, basal ganglia, hippocampus, midbrain, and cerebellum [20,33,34].

PVS are often classified into three types: Type I (basal ganglia), Type II (high convexities/white matter), and Type III (midbrain) [35].

Several visual rating scales exist for estimating PVS burden, but these approaches are time-consuming, subject to observer bias, and challenged by difficulties in differentiating PVS from lacunar infarcts or white matter lesions. Automated segmentation methods are increasingly applied to address these limitations, improving efficiency and reproducibility [36,37].

Dysfunction in PVS-mediated clearance has been linked to the accumulation of neurotoxic proteins such as β-amyloid, tau, α-synuclein, and implicated in conditions including Alzheimer’s disease, Parkinson’s disease, multiple sclerosis, cerebral small vessel disease, and migraine [27,38,39,40,41,42,43,44,45,46,47,48,49].

ePVS prevalence increases with age, reflecting vascular stiffening, endothelial dysfunction, brain atrophy, and chronic low-grade inflammation [49].

In adulthood, ePVS may contribute to neurovascular and neuroinflammatory dysfunction through multiple mechanisms, including disruption of white matter integrity, blood–brain barrier breakdown, increased permeability and inflammation, and impaired glymphatic clearance, all of which may promote cortical disconnection, toxic protein accumulation, and heightened risk of neurodegeneration [50,51].

While the role of PSV in aging and neurodegeneration is increasingly recognized [52,53], its relevance in neurodevelopmental disorders such as ASD remains underexplored. Recent pediatric MRI studies (Table 1) showed a higher prevalence of ePVS in children with ASD, often associated with early brain overgrowth, altered CSF dynamics, and sleep disturbances [54,55,56,57,58]. Whether these alterations persist into adulthood, contribute to neurovascular vulnerability, or could serve as biomarkers of neurobiological aging in ASD remains largely unknown, underscoring a critical gap in lifespan research.

**Table 1:** Key Neuroimaging Findings in ASD.

Modality	Findings in ASD	References
Structural MRI	↑ total brain volume in early childhood; cortical and subcortical overgrowth; regional GM/WM alterations; increased cerebellar volume; pallidum and lateral ventricles enlarged	[9,59,60,61]
DTI/DKI MRI	↓ white matter integrity in long-range tracts including corpus callosum, corona radiata, internal capsule, and inferior longitudinal fasciculus	[8,62,63]
Resting-state fMRI and Structural MRI	altered connectivity in DMN, SN, CEN	[64,65,66]

MRI, magnetic resonance imaging; ↑, increased; GM/WM, grey matter/white matter; ↓, decreased; DTI/DKI MRI, diffusion tensor imaging/diffusion kurtosis imaging magnetic resonance imaging; fMRI, functional magnetic resonance imaging DMN, default mode network; CEN, central executive network; SN, salience network.

## 3. Perivascular Spaces and Autism in Children: What We Know

Research on PVS in autism has so far concentrated on early development, yielding valuable but still incomplete insights into their clinical and neurobiological significance. Early radiological studies identified enlarged ePVS predominantly in the parietal and frontal lobes of people with ASD [67], findings were later corroborated in participants with co-occurring developmental delay [68] and in non-syndromic ASD cohorts [69]. In line with these earlier findings, more recent MRI studies have consistently demonstrated an increased prevalence of ePVS in children with ASD, frequently associated with increased extra-axial CSF (EA-CSF) volumes [54,70,71].

Longitudinal data suggest that PVS alterations may emerge and evolve during infancy. Garic et al. [57] reported progressive PVS enlargement between 6 and 24 months of age in infants later diagnosed with ASD, in parallel with increasing EA-CSF. These findings support hypotheses implicating impaired CSF drainage or immature arachnoid granulations in early PVS dilation [54]. Such dynamics are particularly relevant in the context of concurrent neurovascular remodeling, including white matter expansion, synaptic pruning, and maturation of the glymphatic system, processes fundamental to healthy brain development [72,73]. Disruption may compromise cerebrovascular function, waste clearance, and microstructural integrity, thereby influencing the neurodevelopmental trajectory of ASD.

### 3.1. Evidence on PVS Burden in ASD

Further studies indicate that both PVS volume and visual rating scores are elevated in ASD, particularly among participants with greater clinical severity. Sotgiu et al. [58] reported that white matter-PVS dilation is a characteristic neuroimaging feature in male children, especially the youngest and most severely affected. Extending these findings, Frigerio et al. [74] observed that PVS burden in children with ASD correlates with neurodevelopmental severity and developmental quotient, with a regionally specific pattern characterized by higher PVS counts in frontal regions and lower counts in temporal regions. Wang et al. [8] demonstrated that glymphatic dysfunction co-occurs with enlarged PVS and reduced white matter integrity, particularly in major association tracts such as the corpus callosum, corona radiata, internal capsule, and inferior longitudinal fasciculus—regions critical for network integration. Early myelination deficits in ASD may further impair axonal conduction and neurovascular coupling, thereby hindering CSF transport, reducing glymphatic clearance, and promoting neurotoxic metabolite accumulation, potentially increasing vulnerability to both neurodevelopmental and neurodegenerative processes. Neuropathological evidence supports these findings, implicating microglial clustering, astrocytic endfoot disorganization, and aberrant AQP4 polarization in ASD, changes that may drive glymphatic inefficiency and chronic low-grade neuroinflammation [74]. These cellular abnormalities may not only explain early PVS enlargement but also reflect broader neuroimmune dysregulation.

### 3.2. Functional Implications

Beyond structural enlargement, advanced neuroimaging methods are beginning to reveal functional implications. Diffusion tensor imaging along the perivascular space (DTI-ALPS) and diffusion kurtosis imaging have shown reduced water diffusivity within perivascular pathways in toddlers and young children with ASD, suggesting impaired glymphatic clearance [8,62,63]. This dysfunction may be exacerbated by the exceptionally high prevalence of sleep disturbances in autism [75], given that CSF influx increases by ~60% during sleep [76]. Persistent sleep disruption could amplify clearance deficits, contributing to the accumulation of neurotoxic proteins, including β-amyloid, as documented in histopathological analyses of ASD brains [77,78,79].

Emerging evidence also suggests that PVS abnormalities carry clinical relevance. Specific ePVS distributions, particularly within the rostral middle frontal regions of the dorsolateral prefrontal cortex, have been associated with greater severity of verbal deficits, stereotyped behaviors, and sensory processing abnormalities [58]. Extending this work, Sotgiu et al. [67] demonstrated that PVS enlargement follows a non-random spatial pattern, preferentially aligning with white matter tracts supporting the default mode network, central executive network, and salience network—systems fundamental to social cognition, attentional control, and sensory integration. These spatial associations suggest that PVS alterations may signal localized vulnerabilities within large-scale brain networks central to ASD, rather than representing diffuse or nonspecific vascular changes. An overview of the main findings is provided in Table 2.

In summary, pediatric evidence indicates three key points: (i) PVS changes are detectable in infancy; (ii) they are associated with early brain growth and CSF anomalies; and (iii) they correlate with glymphatic dysfunction and symptom severity. However, longitudinal data beyond childhood remain absent, leaving unresolved whether PVS abnormalities persist, attenuate, or progress into adulthood. Future research should employ optimized 3T MRI protocols, and where available, ultra-high-field MRI (≥7T), together with automated volumetric segmentation, and multimodal approaches (e.g., PET–MRI) to map PVS trajectories across the autistic lifespan and clarify their potential as biomarkers of neurovascular aging.

**Table 2:** Perivascular Spaces and Autism in Children Across Studies.

Study/Year	ASD Population	Main Findings on PVS	Notes
Shen et al. 2018 [55]	High-risk infants (6–24 months)	Increased EA-CSF	Longitudinal study
Li et al. 2022 [63]	Toddlers (24–72 months)	Reduced perivascular diffusivity (DTI/ALPS)	ALPS index reduced in ASD vs. controls; ALPS positively correlated with age (impaired glymphatic function in ASD)
Garic et al. 2023 [57]	Infants (6–24 months)	Increased EA-CSF, ePVS, sleep dysfunction	Longitudinal study
Sotgiu et al. 2023 [58]	Children (2–7 years)	WM-PVS volume associated with male sex, younger age and insomnia in ASD	Link to DLPFC regions [58]; retrospective study
Tian et al. 2025 [64]	Toddlers (24–72 months)	Reduced perivascular diffusivity (DKI-ALPS)	DKI-ALPS may be more sensitive than DTI-ALPS, showing glymphatic impairment correlated with ASD severity
Wang et al. 2025 [8]	Children (3–7 years)	Reduced perivascular diffusivity (aDTI/ALPS)	Retrospective study
Frigerio et al. 2025 [74]	Children (2–8 years)	PVS count and volume associated with severity and developmental quotient	Retrospective study

ASD, Autism Spectrum Disorder; EA-CSF, extra axial-cerebrospinal fluid; ePVS, enlarged perivascular spaces; DTI/ALPS, diffusion tensor imaging along the perivascular spaces; WM-PVS, white matter-perivascular space; DKI-ALPS, diffusion kurtosis imaging analysis along the perivascular space; aDTI/ALPS, automated diffusion tensor imaging along perivascular spaces; DLPFC, dorsolateral prefrontal cortex.

## 4. Perivascular Spaces and Autism in Adulthood: A Neglected Area

Despite growing evidence that PVS alterations may contribute to the neurobiology of ASD during early development, their status in adulthood remains poorly investigated. Whether these early-life changes persist, normalize, or evolve into maladaptive patterns with aging is unknown. This gap is particularly striking given the increasing population of older autistic adults and the recognition of ASD as a lifelong condition requiring biomarkers capable of tracking neurobiological changes across the lifespan. Cognitive profiles in ASD show parallels with typical age-related decline, implicating frontal and medial temporal regions essential for executive function and memory. Evidence of atypical functional connectivity across the lifespan further suggests premature or accelerated cognitive aging in autism, potentially increasing vulnerability to dementia and other neurodegenerative conditions [18]. These concerns align with broader debates on aging trajectories in ASD, for which three theoretical models have been proposed: the Safeguard Theory, the Double Jeopardy Theory and the Parallel Development. The first theory offers a potential explanation for relatively preserved cognitive function in some older autistic patients. It suggests that early compensatory mechanisms protect against neurodegeneration, enhancing neural resilience and reducing the risk of cognitive decline in later life. The second theory argues that aging amplifies pre-existing vulnerabilities in ASD, leading to faster cognitive deterioration. The third theory proposes that autistic and non-autistic adults show comparable rates of age-related cognitive change, but starting from different developmental baselines [80].

Determining which trajectory predominates requires sensitive neurobiological markers; in this context, PVS represents a promising candidate for linking early developmental alterations with aging-related vulnerability. Although recognition of aging in ASD as a clinically and socially relevant issue has increased [81,82,83], neurobiological research on older autistic adults remains scarce; literature reports a paucity of studies, often with modest sample sizes [12,84]. Despite a recent and encouraging increase in studies over the past ten years, the majority of research still focuses primarily on autistic children [85] (Table 3).

Available evidence indicates heightened susceptibility to age-related comorbidities, including metabolic syndrome, cardiovascular disease, sleep disorders, and neurodegenerative conditions [4,86,87,88]—all factors known to impair glymphatic clearance and exacerbate PVS enlargement [24,89].

Several mechanisms may converge to exacerbate PVS abnormalities in autistic adults. Sleep disruption, affecting between 50% and 83% of people with ASD [90,91], reduces CSF-interstitial fluid exchange and metabolic waste clearance [76]. Chronic circadian disruption may therefore promote accumulation of neurotoxic proteins, including amyloid-β and tau, which have been reported in post-mortem analyses of ASD brains [92]. Vascular comorbidities such as hypertension, obesity, and diabetes—more prevalent in autism [87,93]—are strongly linked to cerebral small vessel disease and glymphatic dysfunction [48,94]. In parallel, chronic low-grade inflammation, frequently reported in ASD [95,96], may drive endothelial dysfunction, glial activation, and alterations of perivascular compartments [28].

Importantly, vulnerability to PVS alterations may originate prenatally. Maternal immune activation—including autoimmune disease, metabolic dysregulation, or inflammatory conditions such as asthma—has been associated with increased ASD risk in offspring [97]. Experimental models show that maternal inflammation induces ASD-like behaviors, immune dysregulation, and altered neurodevelopment, suggesting that prenatal exposure may prime the fetal immune system toward long-term neuroimmune abnormalities and impaired glymphatic function [98,99]. Supporting this hypothesis, neuropathological studies in ASD report microglial clustering, astrocytic endfoot disorganization, and aberrant AQP4 polarization [54,100,101], cellular abnormalities are also implicated in glymphatic dysfunction and reduced clearance of amyloid-β and tau in neurodegenerative disorders [27,102].

Importantly, because microglia and astrocytes mediate activity-dependent synaptic pruning and rely on functional perivascular and glymphatic pathways for cytokine and metabolite clearance, disruptions in these pathways may further contribute to the pruning anomalies frequently described in ASD [103]. Despite these converging lines of evidence, direct neuroimaging studies of PVS in autistic adults are virtually absent. Recent advances in ultra-high-field MRI and automated segmentation algorithms [32,104] now enable precise in vivo characterization of PVS morphology, volume, and regional distribution. Integration of these measures with structural, functional, and clinical phenotyping could clarify whether PVS abnormalities in adulthood align with disruptions in large-scale brain networks—such as the default mode, salience, and central executive networks—already implicated in ASD [67,105,106].

**Table 3:** Lifespan View: PVS in Autism.

Life Stage	PVS Imaging Findings	Associated Clinical Features	Unresolved Research Gaps
Infancy (0–2 yrs)	↑ EA-CSF, ePVS [54,56]	Later ASD diagnosis; developmental delays; sleep disturbances	Few longitudinal cohorts; unclear mechanisms
Early childhood (2–6 yrs)	ePVS in basal ganglia and frontal white matter [57,58]; reduced perivascular diffusivity [62,63]	Language/verbal impairments, stereotypies, sensory abnormalities; sleep disturbances	Heterogeneity by sex/severity
Late childhood and adolescence (7–18 yrs)	Higher PVS count in frontal region [73] Alignment of ePVS burden with DMN, CEN, SN networks [66]	Cognitive and executive profiles	Sparse longitudinal tracking
Young–middle adulthood (19–50 yrs)	No imaging studies; indirect evidence from community MRI datasets	Variable cognitive stability; high prevalence of comorbidities	No systematic PVS mapping in adult ASD
Older adulthood (>50–60 yrs)	In general population ↑ PVS [24]; in ASD: no dedicated studies	Potential ↑ risk of cognitive decline and SVD	Virtually no data on PVS in aging autistic adults

yrs, years; EA-CSF, extra axial-cerebrospinal fluid; ASD, Autism Spectrum Disorder; MRI, magnetic resonance imaging; PVS, perivascular spaces; ePVS, enlarged perivascular spaces; DMN, default mode network; CEN, central executive network; SN, salience network; SVD, small vessel disease; ↑, increased.

## 5. Neuroanatomical Hotspots in Autism: Clues for PVS Research

A broad spectrum of neuroanatomical abnormalities has been reported in study participants with ASD compared with neurotypical controls, though findings across neuropathological and neuroimaging studies remain heterogeneous. Reported alterations span several large-scale neural systems, including fronto-temporal and fronto-parietal regions, the amygdala-hippocampal complex, the cerebellum, basal ganglia, and anterior and posterior cingulate cortices; many of these regions overlap with components of the so-called ‘social brain’ network, which subserves social cognition and emotional processing [107].

Early neuropathological investigations suggested megalencephaly as a recurring feature of ASD. Brain size appears slightly reduced at birth but undergoes rapid pathological overgrowth during the first year of life, followed by a marked deceleration between ages 2 and 4 [75]. By adolescence and adulthood, brain volume in most people with ASD converges toward the normative range [108].

The cerebellum has emerged as one of the most consistently implicated structures. Beyond its established role in proprioception and motor coordination, it contributes to higher-order processes, including language, executive functioning, and affective regulation. Its widespread connectivity with limbic, frontal, and temporoparietal regions supports involvement in mentalizing processes [109]. Neuroimaging studies have variably reported increased cerebellar volume [61] and hypoplasia of the central and inferior lobules accompanied by Purkinje cell loss [110]. These changes appear to follow age-related trajectories: reductions in total cerebellar volume, white matter content, Purkinje cell body volume, and region-specific substructures have been described over time [111]. However, systematic lifespan studies are lacking, leaving unresolved how cerebellar alterations in ASD evolve across developmental stages. This gap is particularly relevant given the potential role of PVS in mediating age-related neurovascular changes, as cerebellar regions are tightly integrated with cognitive and affective networks and may show dynamic vulnerability.

The amygdala, a key hub of the limbic system and cortico-striato-thalamo-cortical circuits, integrates multimodal sensory information with higher-order processes underlying social and emotional cognition. The basolateral amygdala links sensory inputs with cortical regions such as the orbitofrontal cortex, anterior cingulate, and medial prefrontal cortex, supporting social cognition and anxiety regulation. Aberrant amygdala development has long been hypothesized as a neural substrate of social impairments in ASD [112]. However, lesion studies in non-human primates emphasize a predominant role in fear responses rather than social behavior per se [113,114]. Given its dense vascularization and integration within limbic circuits, microstructural alterations—potentially reflected in PVS changes—may disrupt connectivity and affective regulation in ASD. Supporting this view, neuroimaging studies consistently reveal functional alterations in autistic people during emotion-processing tasks, particularly within the right posterior fusiform gyrus (fusiform face area, FFA) in the human temporal cortex [115,116]. Although no studies to our knowledge have directly examined PVS alterations within the FFA, the convergence of evidence on enlarged PVS in temporal white matter [32] and altered FFA function in ASD suggests a potential anatomical and physiological overlap. Future multimodal imaging approaches could test whether disrupted glymphatic drainage or perivascular alterations in posterior cortical regions contribute to the atypical development and connectivity of the FFA observed in autism.

While the FFA mediates facial recognition and object expertise, the amygdala plays a critical role in evaluating emotional salience and decoding socially relevant cues, including facial expressions [117], and it receives modulatory input from subcortical structures, including the basal ganglia.

Although there is evidence for basal ganglia volume enlargement in ASD, especially in the pallidum and lateral ventricles [60], and for altered microstructure in the white matter tracts connecting the basal ganglia correlated with repetitive behaviors in children and adolescents [118], data specifically in older autistic adults remain limited. A recent study [62] confirms that many subcortical volumes (including basal ganglia) differ between ASD and neurotypical groups across ages but does not provide definitive insights into trajectories of change in individuals over age 60. Given the basal ganglia’s critical role in motor control, cognitive flexibility, and behavioural regulation, structural and functional alterations in these nuclei could interact with age-related vascular and glymphatic dysfunction in people with ASD, potentially contributing to the heterogeneity of aging trajectories and clinical outcomes.

Importantly, these cortico-striatal-limbic loops extend to the dorsolateral prefrontal cortex (DLPFC), which exerts top-down control over emotion, executive function, and social behaviour. In ASD, the DLPFC shows reduced connectivity with both limbic and subcortical structures [65,66], and recent findings of enlarged PVS in the rostral middle frontal white matter adjacent to the DLPFC [59] raise the possibility that perivascular alterations within these frontostriatal pathways may impair executive and socio-cognitive functions, particularly with aging.

Taken together, these converging lines of evidence support the hypothesis that perivascular alterations across limbic, subcortical, and prefrontal networks may represent a unifying pathophysiological mechanism linking neurovascular health, glymphatic function, and the heterogeneous clinical trajectories of people with autism across the lifespan.

## 6. Future Perspective and Open Questions

Recent advances in neuroimaging and computational analysis provide new opportunities to investigate PVS in both pediatric and adult populations with autism. Although ultra–high-field MRI (≥7 Tesla) offers substantially enhanced spatial resolution and contrast, enabling detection of small PVS that remain invisible on conventional 1.5T or 3T scanners [24,34], 3T MRI remains the most widely available for clinical and research applications. Furthermore, parallel developments in automated segmentation algorithms have improved sensitivity and reproducibility at 3T [104,119]. Together, these innovations facilitate large-scale, cross-sectional, and longitudinal investigations that are essential for delineating how PVS morphology and burden evolve across the autistic lifespan. Moreover, they highlight the importance of including autistic people beyond those frequently followed in hospital settings, as this broader sampling captures milder and less complex presentations as well as more severe forms.

Complementary diffusion MRI approaches tailored to perivascular pathways provide indirect but valuable measures of glymphatic function in vivo [63,120]. Integration with CSF and plasma biomarkers of neuroinflammation and protein clearance (e.g., Aβ, tau, GFAP) may further clarify the clinical significance of PVS abnormalities in autism [121]. Sleep-focused paradigms, such as simultaneous EEG-fMRI or polysomnography combined with high-resolution MRI, are especially promising given the intimate relationship between sleep and glymphatic clearance [70]. Collectively, these multimodal approaches may determine whether PVS represents a stable neuroanatomical trait, a dynamic biomarker of disease progression, or a predictor of later neurovascular or neurodegenerative comorbidities.

The implications extend beyond theoretical relevance. If PVS enlargement reflects impaired glymphatic clearance, chronic low-grade inflammation, or cerebrovascular dysfunction, it could help explain the elevated prevalence of epilepsy, sleep disorders, and possibly neurodegenerative conditions in autism. PVS burden has been linked to cognitive decline and small vessel disease in non-autistic populations [94], raising the possibility of similar mechanisms contributing to age-related vulnerabilities in autistic adults.

From a therapeutic perspective, interventions designed to enhance glymphatic function represent a promising frontier. Sleep optimization—both behavioral and pharmacological—is particularly relevant, given the marked increase in glymphatic activity during slow-wave sleep [77]. Additional strategies, including regular physical activity and management of vascular risk factors, may further support cerebrovascular health and PVS stability [34]. Novel techniques such as focused ultrasound or pharmacological modulation of AQP4 channels, currently under evaluation in other neurological disorders, may ultimately be applicable to autism at the preclinical level, with the potential to guide future clinical developments [103,122].

Advanced MRI protocols may enable systematic monitoring of PVS burden, supporting early identification of individuals at risk for cognitive decline or neurovascular complications. Longitudinal, multimodal studies combining structural MRI, diffusion metrics, and fluid biomarkers are crucial for establishing whether PVS alterations function as stable traits, markers of progression, or predictors of therapeutic response.

Despite these advances, key questions remain unanswered. It is still unknown whether early-life CSF anomalies persist into adulthood or contribute to age-related vulnerabilities in autism. Furthermore, it remains to be clarified if PVS distribution aligns with large-scale brain networks such as the default mode, salience, and central executive systems that represent the symptom core of cognition and behavior in autism.

Beyond passive fluid accumulation, PVS may represent localized alterations in glymphatic function within neural circuits that are critical to ASD, potentially intensified by chronic inflammation or vascular inefficiency. Their spatial overlap with white matter tracts supporting major networks raises the possibility that they could serve as early indicators of neurodegenerative risk, as demonstrated in Alzheimer’s disease and cerebral small vessel disease [123,124].

In summary, current research positions PVS as a promising, but still insufficiently characterized component of autism neurobiology. Given the multidimensional nature of autism, a comprehensive approach that integrates both structural and functional data is essential. Adopting this perspective is critical for fully capturing the disorder’s complexity and for guiding the development of more effective, individualized interventions for autistic people.

## Abbreviations

The following abbreviations are used in this manuscript:

ASD	Autism Spectrum Disorder
ICD-11	International Classification of Diseases
PVS	Perivascular spaces
ePVS	enlarged perivascular spaces
SLYM	subarachnoid lymphatic-like membrane
CSF	cerebrospinal fluid
AQP4	aquaporin 4
MRI	magnetic resonance imaging
fMRI	functional magnetic resonance imaging
EA-CSF	extra-axial CSF
DTI-ALPS	diffusion tensor imaging along the perivascular space
DKI-ALPS	diffusion kurtosis analysis along the perivascular space
DMN	default mode network
CEN	central executive network
SN	salience network

## References

[1] Lord C., Brugha T.S., Charman T., Cusack J., Dumas G., Frazier T., Jones E.J.H., Jones R.M., Pickles A., State M.W. (2020). Autism Spectrum Disorder. Nat. Rev. Dis. Primers.

[2] American Psychiatric Association (2013). Diagnostic and Statistical Manual of Mental Disorders.

[3] Tafolla M., Singer H., Lord C. (2025). Autism Spectrum Disorder Across the Lifespan. Annu. Rev. Clin. Psychol..

[4] Al-Beltagi M. (2021). Autism Medical Comorbidities. WJCP.

[5] Lai M.-C., Kassee C., Besney R., Bonato S., Hull L., Mandy W., Szatmari P., Ameis S.H. (2019). Prevalence of Co-Occurring Mental Health Diagnoses in the Autism Population: A Systematic Review and Meta-Analysis. Lancet Psychiatry.

[6] Loomes R., Hull L., Mandy W.P.L. (2017). What Is the Male-to-Female Ratio in Autism Spectrum Disorder? A Systematic Review and Meta-Analysis. J. Am. Acad. Child Adolesc. Psychiatry.

[7] Bo M., Carta A., Cipriani C., Cavassa V., Simula E.R., Huyen N.T., Phan G.T.H., Noli M., Matteucci C., Sotgiu S. (2024). HERVs Endophenotype in Autism Spectrum Disorder: Human Endogenous Retroviruses, Specific Immunoreactivity, and Disease Association in Different Family Members. Microorganisms.

[8] Wang M., He K., Zhang L., Xu D., Li X., Wang L., Peng B., Qiu A., Dai Y., Zhao C. (2025). Assessment of Glymphatic Function and White Matter Integrity in Children with Autism Using Multi-Parametric MRI and Machine Learning. Eur. Radiol..

[9] Ecker C., Bookheimer S.Y., Murphy D.G.M. (2015). Neuroimaging in Autism Spectrum Disorder: Brain Structure and Function across the Lifespan. Lancet Neurol..

[10] Elsabbagh M., Divan G., Koh Y., Kim Y.S., Kauchali S., Marcín C., Montiel-Nava C., Patel V., Paula C.S., Wang C. (2012). Global Prevalence of Autism and Other Pervasive Developmental Disorders. Autism Res..

[11] Maenner M.J., Warren Z., Williams A.R., Amoakohene E., Bakian A.V., Bilder D.A., Durkin M.S., Fitzgerald R.T., Furnier S.M., Hughes M.M. (2023). Prevalence and Characteristics of Autism Spectrum Disorder Among Children Aged 8 Years—Autism and Developmental Disabilities Monitoring Network, 11 Sites, United States, 2020. MMWR Surveill. Summ..

[12] Bessé M., Morel-Kohlmeyer S., Houy-Durand E., Prévost P., Tuller L., Bouazzaoui B., Taconnat L., Capdeville J., Angel L., Gomot M. (2025). Cognitive and Cerebral Aging Research in Autism: A Systematic Review on an Emerging Topic. Autism Res..

[13] Kris M. (2024). Healthy Aging and Older Adults with Autism: A Scoping Review. Gerontologist.

[14] Van Niekerk M.E.H., Groen W., Vissers C.T.W.M., Van Driel-de Jong D., Kan C.C., Oude Voshaar R.C. (2011). Diagnosing Autism Spectrum Disorders in Elderly People. Int. Psychogeriatr..

[15] Lever A.G., Geurts H.M. (2018). Is Older Age Associated with Higher Self- and Other-Rated ASD Characteristics?. J. Autism Dev. Disord..

[16] Howlin P., Moss P., Savage S., Rutter M. (2013). Social Outcomes in Mid- to Later Adulthood among Individuals Diagnosed with Autism and Average Nonverbal IQ as Children. J. Am. Acad. Child Adolesc. Psychiatry.

[17] Yarar E.Z., Roestorf A., Spain D., Howlin P., Bowler D., Charlton R., Happé F. (2022). Aging and Autism: Do Measures of Autism Symptoms, Co-Occurring Mental Health Conditions, or Quality of Life Differ between Younger and Older Autistic Adults?. Autism Res..

[18] Roestorf A., Bowler D.M., Deserno M.K., Howlin P., Klinger L., McConachie H., Parr J.R., Powell P., Van Heijst B.F.C., Geurts H.M. (2019). “Older Adults with ASD: The Consequences of Aging.” Insights from a Series of Special Interest Group Meetings Held at the International Society for Autism Research 2016–2017. Res. Autism Spectr. Disord..

[19] Edelson S.M., Nicholas D.B., Stoddart K.P., Bauman M.B., Mawlam L., Lawson W.B., Jose C., Morris R., Wright S.D. (2021). Strategies for Research, Practice, and Policy for Autism in Later Life: A Report from a Think Tank on Aging and Autism. J. Autism Dev. Disord..

[20] Rudie J.D., Rauschecker A.M., Nabavizadeh S.A., Mohan S. (2018). Neuroimaging of Dilated Perivascular Spaces: From Benign and Pathologic Causes to Mimics. J. Neuroimaging.

[21] Lynch K.M., Sepehrband F., Toga A.W., Choupan J. (2023). Brain Perivascular Space Imaging across the Human Lifespan. NeuroImage.

[22] Kipnis J. (2024). The Anatomy of Brainwashing. Science.

[23] Szczygielski J., Kopańska M., Wysocka A., Oertel J. (2021). Cerebral Microcirculation, Perivascular Unit, and Glymphatic System: Role of Aquaporin-4 as the Gatekeeper for Water Homeostasis. Front. Neurol..

[24] Wardlaw J.M., Benveniste H., Nedergaard M., Zlokovic B.V., Mestre H., Lee H., Doubal F.N., Brown R., Ramirez J., MacIntosh B.J. (2020). Perivascular Spaces in the Brain: Anatomy, Physiology and Pathology. Nat. Rev. Neurol..

[25] Møllgård K., Beinlich F.R.M., Kusk P., Miyakoshi L.M., Delle C., Plá V., Hauglund N.L., Esmail T., Rasmussen M.K., Gomolka R.S. (2023). A Mesothelium Divides the Subarachnoid Space into Functional Compartments. Science.

[26] Plá V., Bitsika S., Giannetto M.J., Ladron-de-Guevara A., Gahn-Martinez D., Mori Y., Nedergaard M., Møllgård K. (2023). Structural Characterization of SLYM—A 4th Meningeal Membrane. Fluids Barriers CNS.

[27] Iliff J.J., Wang M., Liao Y., Plogg B.A., Peng W., Gundersen G.A., Benveniste H., Vates G.E., Deane R., Goldman S.A. (2012). A Paravascular Pathway Facilitates CSF Flow Through the Brain Parenchyma and the Clearance of Interstitial Solutes, Including Amyloid β. Sci. Transl. Med..

[28] Hablitz L.M., Nedergaard M. (2021). The Glymphatic System: A Novel Component of Fundamental Neurobiology. J. Neurosci..

[29] Murdock M.H., Yang C.-Y., Sun N., Pao P.-C., Blanco-Duque C., Kahn M.C., Kim T., Lavoie N.S., Victor M.B., Islam M.R. (2024). Multisensory Gamma Stimulation Promotes Glymphatic Clearance of Amyloid. Nature.

[30] Mestre H., Hablitz L.M., Xavier A.L., Feng W., Zou W., Pu T., Monai H., Murlidharan G., Castellanos Rivera R.M., Simon M.J. (2018). Aquaporin-4-Dependent Glymphatic Solute Transport in the Rodent Brain. eLife.

[31] Plog B.A., Dashnaw M.L., Hitomi E., Peng W., Liao Y., Lou N., Deane R., Nedergaard M. (2015). Biomarkers of Traumatic Injury Are Transported from Brain to Blood via the Glymphatic System. J. Neurosci..

[32] Barisano G., Lynch K.M., Sibilia F., Lan H., Shih N.-C., Sepehrband F., Choupan J. (2022). Imaging Perivascular Space Structure and Function Using Brain MRI. NeuroImage.

[33] Yu L., Hu X., Li H., Zhao Y. (2022). Perivascular Spaces, Glymphatic System and MR. Front. Neurol..

[34] Barisano G., Sheikh-Bahaei N., Law M., Toga A.W., Sepehrband F. (2021). Body Mass Index, Time of Day and Genetics Affect Perivascular Spaces in the White Matter. J. Cereb. Blood Flow Metab..

[35] Potter G.M., Chappell F.M., Morris Z., Wardlaw J.M. (2015). Cerebral Perivascular Spaces Visible on Magnetic Resonance Imaging: Development of a Qualitative Rating Scale and Its Observer Reliability. Cerebrovasc. Dis..

[36] Kwee R.M., Kwee T.C. (2007). Virchow-Robin Spaces at MR Imaging. RadioGraphics.

[37] Botta D., Hutuca I., Ghoul E.E., Sveikata L., Assal F., Lövblad K.-O., Kurz F.T. (2025). Emerging Non-Invasive MRI Techniques for Glymphatic System Assessment in Neurodegenerative Disease. J. Neuroradiol..

[38] Xin S.H., Tan L., Cao X., Yu J.T., Tan L. (2018). Clearance of Amyloid Beta and Tau in Alzheimer’s Disease: From Mechanisms to Therapy. Neurotox. Res..

[39] Peng W., Achariyar T.M., Li B., Liao Y., Mestre H., Hitomi E., Regan S., Kasper T., Peng S., Ding F. (2016). Suppression of Glymphatic Fluid Transport in a Mouse Model of Alzheimer’s Disease. Neurobiol. Dis..

[40] Li H., Yao Q., Huang X., Yang X., Yu C. (2024). The Role and Mechanism of Aβ Clearance Dysfunction in the Glymphatic System in Alzheimer’s Disease Comorbidity. Front. Neurol..

[41] Lopes D.M., Llewellyn S.K., Bury S.E., Wang J., Wells J.A., Gegg M.E., Verona G., Lythgoe M.F., Harrison I.F. (2025). The Influence of the Glymphatic System on α-Synuclein Propagation: The Role of Aquaporin-4. Brain.

[42] Schubert J.J., Veronese M., Marchitelli L., Bodini B., Tonietto M., Stankoff B., Brooks D.J., Bertoldo A., Edison P., Turkheimer F.E. (2019). Dynamic 11C-PiB PET Shows Cerebrospinal Fluid Flow Alterations in Alzheimer Disease and Multiple Sclerosis. J. Nucl. Med..

[43] Reeves B.C., Karimy J.K., Kundishora A.J., Mestre H., Cerci H.M., Matouk C., Alper S.L., Lundgaard I., Nedergaard M., Kahle K.T. (2020). Glymphatic System Impairment in Alzheimer’s Disease and Idiopathic Normal Pressure Hydrocephalus. Trends Mol. Med..

[44] Shen T., Yue Y., Ba F., He T., Tang X., Hu X., Pu J., Huang C., Lv W., Zhang B. (2022). Diffusion along Perivascular Spaces as Marker for Impairment of Glymphatic System in Parkinson’s Disease. NPJ Park. Dis..

[45] Zhang Y., Zhang C., He X.Z., Li Z.H., Meng J.C., Mao R.T., Li X., Xue R., Gui Q., Zhang G.X. (2023). Interaction Between the Glymphatic System and α-Synuclein in Parkinson’s Disease. Mol. Neurobiol..

[46] Granberg T., Moridi T., Brand J.S., Neumann S., Hlavica M., Piehl F., Ineichen B.V. (2020). Enlarged Perivascular Spaces in Multiple Sclerosis on Magnetic Resonance Imaging: A Systematic Review and Meta-Analysis. J. Neurol..

[47] Alghanimy A., Work L.M., Holmes W.M. (2024). The Glymphatic System and Multiple Sclerosis: An Evolving Connection. Mult. Scler. Relat. Disord..

[48] Ang P.S., Zhang D.M., Azizi S.-A., Norton De Matos S.A., Brorson J.R. (2024). The Glymphatic System and Cerebral Small Vessel Disease. J. Stroke Cerebrovasc. Dis..

[49] Vittorini M.G., Sahin A., Trojan A., Yusifli S., Alashvili T., Bonifácio G.V., Paposhvili K., Tischler V., Lampl C., Sacco S. (2024). The Glymphatic System in Migraine and Other Headaches. J. Headache Pain.

[50] Lara F.R., Scruton A.L., Pinheiro A., Demissie S., Parva P., Charidimou A., Francis M., Himali J.J., DeCarli C., Beiser A. (2022). Aging, Prevalence and Risk Factors of MRI-Visible Enlarged Perivascular Spaces. Aging.

[51] Ineichen B.V., Okar S.V., Proulx S.T., Engelhardt B., Lassmann H., Reich D.S. (2022). Perivascular Spaces and Their Role in Neuroinflammation. Neuron.

[52] Shulyatnikova T., Hayden M.R. (2023). Why Are Perivascular Spaces Important?. Medicina.

[53] Benveniste H., Liu X., Koundal S., Sanggaard S., Lee H., Wardlaw J. (2019). The Glymphatic System and Waste Clearance with Brain Aging: A Review. Gerontology.

[54] Xiong Y., Yu Q., Zhi H., Peng H., Xie M., Li R., Li K., Ma Y., Sun P. (2024). Advances in the Study of the Glymphatic System and Aging. CNS Neurosci. Ther..

[55] Shen M.D., Nordahl C.W., Li D.D., Lee A., Angkustsiri K., Emerson R.W., Rogers S.J., Ozonoff S., Amaral D.G. (2018). Extra-Axial Cerebrospinal Fluid in High-Risk and Normal-Risk Children with Autism Aged 2–4 Years: A Case-Control Study. Lancet Psychiatry.

[56] Peterson M., Prigge M.B.D., Bigler E.D., Zielinski B., King J.B., Lange N., Alexander A., Lainhart J.E., Nielsen J.A. (2021). Evidence for Normal Extra-Axial Cerebrospinal Fluid Volume in Autistic Males from Middle Childhood to Adulthood. NeuroImage.

[57] Garic D., McKinstry R.C., Rutsohn J., Slomowitz R., Wolff J., MacIntyre L.C., Weisenfeld L.A.H., Kim S.H., Pandey J., John T. (2023). Enlarged Perivascular Spaces in Infancy and Autism Diagnosis, Cerebrospinal Fluid Volume, and Later Sleep Problems. JAMA Netw. Open.

[58] Sotgiu M.A., Lo Jacono A., Barisano G., Saderi L., Cavassa V., Montella A., Crivelli P., Carta A., Sotgiu S. (2023). Brain Perivascular Spaces and Autism: Clinical and Pathogenic Implications from an Innovative Volumetric MRI Study. Front. Neurosci..

[59] Sotgiu S., Cavassa V., Puci M.V., Sotgiu M.A., Turilli D., Jacono A.L., Nuvoli A., Masala S., Barisano G., Carta A. (2025). Enlarged Perivascular Spaces under the Dorso-Lateral Prefrontal Cortex and Severity of Autism. Sci. Rep..

[60] Turner A.H., Greenspan K.S., Van Erp T.G.M. (2016). Pallidum and Lateral Ventricle Volume Enlargement in Autism Spectrum Disorder. Psychiatry Res. Neuroimaging.

[61] Baizer J.S. (2024). Neuroanatomy of Autism: What Is the Role of the Cerebellum?. Cereb. Cortex.

[62] Christensen D., Shin Y.S., Wang J., Cuomo C.R., Dentry T., Gemmell H.M., Pulver S.L., Orlando A.-M., McKinney W.S., Stevens C.J. (2025). Subcortical Brain Volume Variations in Autistic Individuals across the Lifespan. Mol. Autism.

[63] Li X., Ruan C., Zibrila A.I., Musa M., Wu Y., Zhang Z., Liu H., Salimeen M. (2022). Children with Autism Spectrum Disorder Present Glymphatic System Dysfunction Evidenced by Diffusion Tensor Imaging along the Perivascular Space. Medicine.

[64] Tian P., Xue Y., Zhu X., Liu Z., Bian B., Jia F., Dou L., Lv X., Zhao T., Li D. (2025). Abnormal Glymphatic System Function in Children with Autism Spectrum Disorder: A Diffusion Kurtosis Imaging Study. Eur. J. Pediatr..

[65] Uddin L.Q., Supekar K., Menon V. (2013). Reconceptualizing Functional Brain Connectivity in Autism from a Developmental Perspective. Front. Hum. Neurosci..

[66] Courchesne E., Pierce K. (2005). Brain Overgrowth in Autism during a Critical Time in Development: Implications for Frontal Pyramidal Neuron and Interneuron Development and Connectivity. Int. J. Dev. Neurosci..

[67] Sotgiu S., Barisano G., Cavassa V., Puci M.V., Nuvoli A., Masala S.A., Carta A. (2025). Cognitive Brain Networks and Enlarged Perivascular Spaces: Implications for Symptom Severity and Support Needs in Children with Autism. J. Clin. Med..

[68] Taber K.H., Shaw J.B., Loveland K.A., Pearson D.A., Lane D.M., Hayman L.A. (2004). Accentuated Virchow-Robin Spaces in the Centrum Semiovale in Children with Autistic Disorder. J. Comput. Assist. Tomogr..

[69] Zeegers M., Van Der Grond J., Durston S., Nievelstein R.J., Witkamp T., Van Daalen E., Buitelaar J., Engeland H.V. (2006). Radiological Findings in Autistic and Developmentally Delayed Children. Brain Dev..

[70] Boddaert N., Zilbovicius M., Philipe A., Robel L., Bourgeois M., Barthélemy C., Seidenwurm D., Meresse I., Laurier L., Desguerre I. (2009). MRI Findings in 77 Children with Non-Syndromic Autistic Disorder. PLoS ONE.

[71] Shen M.D., Nordahl C.W., Young G.S., Wootton-Gorges S.L., Lee A., Liston S.E., Harrington K.R., Ozonoff S., Amaral D.G. (2013). Early Brain Enlargement and Elevated Extra-Axial Fluid in Infants Who Develop Autism Spectrum Disorder. Brain.

[72] Shen M.D., Kim S.H., McKinstry R.C., Gu H., Hazlett H.C., Nordahl C.W., Emerson R.W., Shaw D., Elison J.T., Swanson M.R. (2017). Increased Extra-Axial Cerebrospinal Fluid in High-Risk Infants Who Later Develop Autism. Biol. Psychiatry.

[73] Rasmussen M.K., Mestre H., Nedergaard M. (2022). Fluid Transport in the Brain. Physiol. Rev..

[74] Frigerio G., Rizzato G., Peruzzo D., Ciceri T., Mani E., Lanteri F., Mariani V., Molteni M., Agarwal N. (2025). Perivascular Space Burden in Children with Autism Spectrum Disorder Correlates With Neurodevelopmental Severity. J. Magn. Reson. Imaging.

[75] Fetit R., Hillary R.F., Price D.J., Lawrie S.M. (2021). The Neuropathology of Autism: A Systematic Review of Post-Mortem Studies of Autism and Related Disorders. Neurosci. Biobehav. Rev..

[76] Carmassi C., Palagini L., Caruso D., Masci I., Nobili L., Vita A., Dell’Osso L. (2019). Systematic Review of Sleep Disturbances and Circadian Sleep Desynchronization in Autism Spectrum Disorder: Toward an Integrative Model of a Self-Reinforcing Loop. Front. Psychiatry.

[77] Xie L., Kang H., Xu Q., Chen M.J., Liao Y., Thiyagarajan M., O’Donnell J., Christensen D.J., Nicholson C., Iliff J.J. (2013). Sleep Drives Metabolite Clearance from the Adult Brain. Science.

[78] Wegiel J., Frackowiak J., Mazur-Kolecka B., Schanen N.C., Cook E.H., Sigman M., Brown W.T., Kuchna I., Wegiel J., Nowicki K. (2012). Abnormal Intracellular Accumulation and Extracellular Aβ Deposition in Idiopathic and Dup15q11.2-Q13 Autism Spectrum Disorders. PLoS ONE.

[79] Westmark C.J. (2013). What’s hAPPening at Synapses? The Role of Amyloid β-Protein Precursor and β-Amyloid in Neurological Disorders. Mol. Psychiatry.

[80] Westmark C.J., Sokol D.K., Maloney B., Lahiri D.K. (2016). Novel Roles of Amyloid-Beta Precursor Protein Metabolites in Fragile X Syndrome and Autism. Mol. Psychiatry.

[81] Wang J., Christensen D., Coombes S.A., Wang Z. (2024). Cognitive and Brain Morphological Deviations in Middle-to-Old Aged Autistic Adults: A Systematic Review and Meta-Analysis. Neurosci. Biobehav. Rev..

[82] Happé F., Charlton R.A. (2012). Aging in Autism Spectrum Disorders: A Mini-Review. Gerontology.

[83] Hategan A., Bourgeois J.A., Goldberg J. (2017). Aging with Autism Spectrum Disorder: An Emerging Public Health Problem. Int. Psychogeriatr..

[84] Mason D., Ronald A., Ambler A., Caspi A., Houts R., Poulton R., Ramrakha S., Wertz J., Moffitt T.E., Happé F. (2021). Autistic Traits Are Associated with Faster Pace of Aging: Evidence from the Dunedin Study at Age 45. Autism Res..

[85] Lever A.G., Geurts H.M. (2016). Age-Related Differences in Cognition across the Adult Lifespan in Autism Spectrum Disorder. Autism Res..

[86] Mason D., Stewart G.R., Capp S.J., Happé F. (2022). Older Age Autism Research: A Rapidly Growing Field, but Still a Long Way to Go. Autism Adulthood.

[87] Croen L.A., Zerbo O., Qian Y., Massolo M.L., Rich S., Sidney S., Kripke C. (2015). The Health Status of Adults on the Autism Spectrum. Autism.

[88] Bishop L., Charlton R.A., McLean K.J., McQuaid G.A., Lee N.R., Wallace G.L. (2023). Cardiovascular Disease Risk Factors in Autistic Adults: The Impact of Sleep Quality and Antipsychotic Medication Use. Autism Res..

[89] Vivanti G., Tao S., Lyall K., Robins D.L., Shea L.L. (2021). The Prevalence and Incidence of Early-Onset Dementia among Adults with Autism Spectrum Disorder. Autism Res..

[90] Jessen N.A., Munk A.S.F., Lundgaard I., Nedergaard M. (2015). The Glymphatic System: A Beginner’s Guide. Neurochem. Res..

[91] Ballester P., Richdale A.L., Baker E.K., Peiró A.M. (2020). Sleep in Autism: A Biomolecular Approach to Aetiology and Treatment. Sleep Med. Rev..

[92] Rhodus E.K., Barber J., Kryscio R.J., Abner E.L., Bahrani A.A., Lewis K.E.S., Carey B., Nelson P.T., Van Eldik L.J., Jicha G.A. (2022). Frontotemporal Neurofibrillary Tangles and Cerebrovascular Lesions Are Associated with Autism Spectrum Behaviors in Late-Life Dementia. J. Neurol..

[93] Hand B.N., Angell A.M., Harris L., Carpenter L.A. (2020). Prevalence of Physical and Mental Health Conditions in Medicare-Enrolled, Autistic Older Adults. Autism.

[94] Wardlaw J.M., Smith C., Dichgans M. (2019). Small Vessel Disease: Mechanisms and Clinical Implications. Lancet Neurol..

[95] Estes M.L., McAllister A.K. (2015). Immune Mediators in the Brain and Peripheral Tissues in Autism Spectrum Disorder. Nat. Rev. Neurosci..

[96] Sotgiu S., Manca S., Gagliano A., Minutolo A., Melis M.C., Pisuttu G., Scoppola C., Bolognesi E., Clerici M., Guerini F.R. (2020). Immune Regulation of Neurodevelopment at the Mother–Foetus Interface: The Case of Autism. Clin. Transl. Immunol..

[97] Gardner R.M., Brynge M., Sjöqvist H., Dalman C., Karlsson H. (2025). Maternal Immune Activation and Autism in Offspring: What Is the Evidence for Causation?. Biol. Psychiatry.

[98] Hughes H.K., Moreno R.J., Ashwood P. (2023). Innate Immune Dysfunction and Neuroinflammation in Autism Spectrum Disorder (ASD). Brain Behav. Immun..

[99] Careaga M., Murai T., Bauman M.D. (2017). Maternal Immune Activation and Autism Spectrum Disorder: From Rodents to Nonhuman and Human Primates. Biol. Psychiatry.

[100] Vargas D.L., Nascimbene C., Krishnan C., Zimmerman A.W., Pardo C.A. (2005). Neuroglial Activation and Neuroinflammation in the Brain of Patients with Autism. Ann. Neurol..

[101] Zeidán-Chuliá F., Salmina A.B., Malinovskaya N.A., Noda M., Verkhratsky A., Moreira J.C.F. (2014). The Glial Perspective of Autism Spectrum Disorders. Neurosci. Biobehav. Rev..

[102] Mestre H., Mori Y., Nedergaard M. (2020). The Brain’s Glymphatic System: Current Controversies. Trends Neurosci..

[103] Unnisa A., Greig N.H., Kamal M.A. (2023). Modelling the Interplay Between Neuron-Glia Cell Dysfunction and GlialTherapy in Autism Spectrum Disorder. Curr. Neuropharmacol..

[104] Sepehrband F., Barisano G., Sheikh-Bahaei N., Cabeen R.P., Choupan J., Law M., Toga A.W. (2019). Image Processing Approaches to Enhance Perivascular Space Visibility and Quantification Using MRI. Sci. Rep..

[105] Uddin L.Q., Supekar K., Lynch C.J., Khouzam A., Phillips J., Feinstein C., Ryali S., Menon V. (2013). Salience Network–Based Classification and Prediction of Symptom Severity in Children with Autism. JAMA Psychiatry.

[106] Sato W., Uono S. (2019). The atypical social brain network in autism: Advances in structural and functional MRI studies. Curr. Opin. Neurol..

[107] Berg L.M., Gurr C., Leyhausen J., Seelemeyer H., Bletsch A., Schaefer T., Pretzsch C.M., Oakley B., Loth E., Floris D.L. (2023). The Neuroanatomical Substrates of Autism and ADHD and Their Link to Putative Genomic Underpinnings. Mol. Autism.

[108] Redcay E., Courchesne E. (2005). When Is the Brain Enlarged in Autism? A Meta-Analysis of All Brain Size Reports. Biol. Psychiatry.

[109] Clausi S., Olivito G., Siciliano L., Lupo M., Laghi F., Baiocco R., Leggio M. (2021). The Cerebellum Is Linked to Theory of Mind Alterations in Autism. A Direct Clinical and MRI Comparison between Individuals with Autism and Cerebellar Neurodegenerative Pathologies. Autism Res..

[110] Donovan A.P.A., Basson M.A. (2017). The Neuroanatomy of Autism—A Developmental Perspective. J. Anat..

[111] McElroy C.L., Wang B., Zhang H., Jin K. (2024). Cerebellum and Aging: Update and Challenges. Aging Dis..

[112] Baron-Cohen S., Ring H.A., Bullmore E.T., Wheelwright S., Ashwin C., Williams S.C.R. (2000). The Amygdala Theory of Autism. Neurosci. Biobehav. Rev..

[113] Amaral D.G., Schumann C.M., Nordahl C.W. (2008). Neuroanatomy of Autism. Trends Neurosci..

[114] Seguin D., Pac S., Wang J., Nicolson R., Martinez-Trujillo J., Anagnostou E., Lerch J.P., Hammill C., Schachar R., Crosbie J. (2022). Amygdala Subnuclei Volumes and Anxiety Behaviors in Children and Adolescents with Autism Spectrum Disorder, Attention Deficit Hyperactivity Disorder, and Obsessive–Compulsive Disorder. Hum. Brain Mapp..

[115] Liu J., Chen H., Wang H., Wang Z. (2025). Neural Correlates of Facial Recognition Deficits in Autism Spectrum Disorder: A Comprehensive Review. Front. Psychiatry.

[116] Floris D.L., Llera A., Zabihi M., Moessnang C., Jones E.J.H., Mason L., Haartsen R., Holz N.E., Mei T., Elleaume C. (2025). A Multimodal Neural Signature of Face Processing in Autism within the Fusiform Gyrus. Nat. Mental Health.

[117] Meyer-Lindenberg H., Moessnang C., Oakley B., Ahmad J., Mason L., Jones E.J.H., Hayward H.L., Cooke J., Crawley D., Holt R. (2022). Facial Expression Recognition Is Linked to Clinical and Neurofunctional Differences in Autism. Mol. Autism.

[118] Wilkes B.J., Archer D.B., Farmer A.L., Bass C., Korah H., Vaillancourt D.E., Lewis M.H. (2024). Cortico-Basal Ganglia White Matter Microstructure Is Linked to Restricted Repetitive Behavior in Autism Spectrum Disorder. Mol. Autism.

[119] Boespflug E.L., Schwartz D.L., Lahna D., Pollock J., Iliff J.J., Kaye J.A., Rooney W., Silbert L.C. (2018). MR Imaging–Based Multimodal Autoidentification of Perivascular Spaces (mMAPS): Automated Morphologic Segmentation of Enlarged Perivascular Spaces at Clinical Field Strength. Radiology.

[120] Taoka T., Naganawa S. (2020). Glymphatic Imaging Using MRI. J. Magn. Reson. Imaging.

[121] Rasmussen M.K., Mestre H., Nedergaard M. (2018). The Glymphatic Pathway in Neurological Disorders. Lancet Neurol..

[122] Davoudi S., Rahdar M., Hosseinmardi N., Behzadi G., Janahmadi M. (2023). Chronic Inhibition of Astrocytic Aquaporin-4 Induces Autistic-like Behavior in Control Rat Offspring Similar to Maternal Exposure to Valproic Acid. Physiol. Behav..

[123] Banerjee G., Kim H.J., Fox Z., Jäger H.R., Wilson D., Charidimou A., Na H.K., Na D.L., Seo S.W., Werring D.J. (2017). MRI-Visible Perivascular Space Locations Distinguishes Alzheimer’s Disease from Subcortical Vascular Cognitive Impairment Independently of Amyloid Burden. Brain.

[124] Brown R., Benveniste H., Black S.E., Charpak S., Dichgans M., Joutel A., Nedergaard M., Smith K.J., Zlokovic B.V., Wardlaw J.M. (2018). Understanding the Role of the Perivascular Space in Cerebral Small Vessel Disease. Cardiovasc. Res..

